# Sanctuary to sinkhole and back again: video gaming and esport as a transition environment for young people at risk of marginalization

**DOI:** 10.3389/fpsyg.2026.1793190

**Published:** 2026-04-16

**Authors:** Michael Geoffrey Trotter, Lukas Linnér, Kalle Jonasson, Jolan Kegelaers, Matthew Watson, Lars Domino Østergaard, Daniel Kjeldgaard Larsen, Jon Brain, Cleo Schyvinck, Michael Gill, Karl Sergel, Håkan Damm, Martin Johansson, Mads Arnborg Didriksen, André Baumann, Niklas Cederström, Urban Johnson

**Affiliations:** 1 School of Health and Welfare, Halmstad University, Halmstad, Sweden; 2 Faculty of Psychology and Educational Sciences, Vrije Universiteit Brussel, Brussels, Belgium; 3 Department of Performance Psychology, German Sport University Cologne, Cologne, Germany; 4 Department of Health Science and Technology, Aalborg University, Aalborg, Denmark; 5 KFUM's Sociale Arbejde (YMCA Social Work), Middelfart, Denmark; 6 Thomas More University of Applied Sciences, Antwerp, Belgium; 7 RF-SISU Västerbotten (Riksidrottsförbundet och SISU Idrottsutbildarna, Distrikt Västerbotten), Umeå, Sweden; 8 Fuzed AB, Stockholm, Sweden; 9 Ystads Idrottsförening Handbollsförening (Ystads IF HF), Ystad, Sweden; 10 Gymnasieskolan Knut Hahn Ronneby Kommun, Ronneby, Sweden; 11 Dansk Gymnastik and Idrætsforening, Bredsten, Denmark; 12 Bredde-e-sport Alliansen, Oslo, Norway

**Keywords:** education, esports, gaming, marginalization, NEET, youth work

## The marginalization staircase as a socioeconomic and humanitarian problem

Evidence shows that youth NEET (“Not in Education, Employment or Training”) status is associated with substantial socio-economic costs and elevated risk of physical and mental ill health (Lindblad et al., 2024; Rahmani et al., 2024). In Europe alone, approximately 12.6% of young people are categorized as NEET, which carries an estimated economic burden exceeding €100 billion annually and has accumulated to over €1.6 trillion over the past decade (Eurostat, 2025; Mascherini, 2025). In Sweden, youth school absenteeism alone has been estimated to cost over approximately 14-24 billion Euro annually and is linked to a downward spiral into NEET status and social isolation, described as the “marginalization staircase” (Nilsson Lundmark and Nilsson, 2022). The marginalization staircase conceptualizes disengagement as a stepwise and cumulative process, in which consequences become more extensive, societal costs increase exponentially, and the likelihood of reversing marginalization diminishes as young people move further along the trajectory. At each step, progression along the staircase reflects the cumulative impact of individual vulnerabilities interacting with educational and social environments, progressively weakening engagement with school, work, and community life. Longitudinal and population-based studies indicate that mental health problems and modifiable adolescent psychosocial factors, such as low self-esteem, external locus of control, low

physical activity, limited job aspirations and negative attitudes to school, precede and predict later NEET outcomes (Gariépy et al., 2022; Lindblad et al., 2024; Tayfur et al., 2022). Evidence further suggests that neurodivergent (e.g., ADHD and Autism) children and adolescents are disproportionately represented among those entering disengagement trajectories, particularly when mental health difficulties coincide with repeated experiences of school failure and social rejection (Hilding et al., 2025; Lindblad et al., 2024). Young people who become NEET show higher levels of depression, anxiety and suicidal ideation and face increased risk of long-term unemployment and social exclusion (Gariépy et al., 2022; UNICEF, 2021). Importantly, economic evaluations indicate that early, preventive youth interventions typically generate returns of approximately three to seven times the initial public investment, reflecting the substantial costs avoided when disengagement is addressed before it becomes entrenched (Frontier Economics, 2022; Nilsson Lundmark and Nilsson, 2022). Taken together, this body of work underscores that NEET status cannot be understood solely as the product of isolated individual deficits, but rather as the outcome of a cumulative misalignment between young people and the educational and social environments intended to support them, highlighting the urgency of preventive action early on the marginalization staircase.

## Unmet psychological needs as a driving mechanism of disengagement

For many children and adolescents, video gaming may function as a coping mechanism or “sanctuary” where core psychological needs (i.e., autonomy, competence and relatedness; Ryan and Deci, 2000) are more reliably satisfied than in offline settings. A central driver of this reliance appears to be the persistent frustration of basic psychological needs in offline contexts such as educational and social environments. Self-Determination Theory posits that wellbeing and motivation depend on the satisfaction of competence (i.e., perceived competence or self-efficacy), autonomy (i.e., an internal perceived locus of causality) and relatedness (i.e., perceived belonging and connectedness with others) (Ryan and Deci, 2000), and that chronic frustration of these needs is associated with withdrawal and disengagement (Allen and Anderson, 2018). Research suggests that video games strongly support needs for competence, autonomy and relatedness, and that this need satisfaction underpins enjoyment, intrinsic motivation and sustained engagement in play (Przybylski et al., 2010). In contrast, educational contexts may undermine these needs through repeated experiences of underachievement, learning difficulties, bullying, social exclusion, or controlling, unsupportive or abusive teacher behaviors (Schyvinck et al., 2024). Such experiences have been shown to predict increased need frustration, school refusal (an “attendance problem that manifests in various ways, such as: not attending school for a long time; not staying in class all the time; arriving late to school; and students attending school only because they are forced to by their parents”; Filippello et al., 2019, p. 1) and truancy (Nouwen and Clycq, 2019).

Evidence indicates that when real-world need satisfaction is low but in-game need satisfaction is high, symptoms of disordered gaming are elevated and wellbeing is poorer, consistent with a “need-density” process in which games compensate for, but do not resolve, deficits in everyday life (Allen and Anderson, 2018). Under conditions of sustained failure or rejection, disengagement from school may function as an avoidance-based coping response aimed at preserving self-worth by limiting exposure to further threat or failure (Melodia et al., 2022). Recent qualitative research with Swedish neurodivergent youth similarly describes games and game culture as central sources of recovery, emotion regulation and social contact for young people who often feel exhausted by school demands and excluded from traditional associations (Hilding et al., 2025). Children and neurodivergent adolescents appear particularly exposed to such need frustration in school environments and may therefore be more likely to seek alternative contexts that fulfill their basic psychological needs.

Solitary and unstructured video gaming is associated with poorer wellbeing, more gaming-related problems if it primary way to cope with the issues in one's life (Beres et al., 2023; Lee and Chen, 2023). Such patterns of engagement also appear unlikely to foster the emotion- and self-regulation capacities needed to manage stress and pursue long-term goals in education, work, and relationships (Formosa et al., 2025; Trotter et al., 2021). Under conditions of sustained need frustration, compensatory engagement in gaming or esports may evolve into a “sinkhole” that displaces other important life activities and reduces exposure to environments that could support broader psychological development. In addition, unstructured and poorly supervised gaming and esports environments may expose some young people to harmful behaviors perpetrated by their peers either online or offline such as interpersonal violence (sexual, physical, psychological, neglect), and discrimination which can further undermine wellbeing and social participation (Brehm, 2013; Breuer et al., 2015; Fox and Tang, 2014, 2017). Together, these processes can further reduce school attendance, limit social participation and reinforce withdrawal, accelerating movement along the marginalization staircase rather than resolving it (Allen and Anderson, 2018; Hilding et al., 2025; Nouwen and Clycq, 2019). In turn, this also highlights the importance of robust safeguarding policies, practices and structures that intentionally counter the risks of solitary and unstructured gaming through clear norms, trusted adult presence, trained professionals, and appropriate mechanisms for recognizing and addressing safety concerns.

## Transition environments and gaming-based educational interventions

Socioeconomic analyses of Swedish youth highlight that leader-led, regular and organized structured leisure is associated with better physical and mental health, greater school satisfaction and improved grades, though evidence suggests that unstructured leisure time (e.g., socializing with peers), may be linked to poorer outcomes and heightened risk of exclusion (Mahoney and Stattin, 2000; Nilsson Lundmark and Nilsson, 2022). Youth clubs (e.g., community clubs or sports clubs) are identified as key preventive arenas, particularly for young people already on the margins of school and community life, and early investment in such settings yields substantial long-term savings by reducing crime, unemployment and health service use (Frontier Economics, 2022; Nilsson Lundmark and Nilsson, 2022). Internationally based organizations have identified that at the European level, promoting and protecting adolescent mental health requires safe everyday environments that offer connection, support and opportunities to play, learn and grow, not only clinical services (UNICEF, 2021).

Recent work in sport and exercise psychology provides a more detailed conceptualization of how transition environments could function during periods of change (Henriksen et al., 2024). Drawing on ecological systems perspectives, transition environments are described as temporary and dynamic systems designed to bridge contexts by reducing immediate pressures while maintaining engagement, safety, and developmental continuity (Henriksen et al., 2024). Ecological approaches to athletes' careers describe transition environments as systems that bridge a donor setting and a receiving setting, linking key actors in the individual's life across home, education and organized activity, and preventing transitions from becoming crises through coordinated support across settings (Henriksen et al., 2024). Case studies of successful dual career development environments show that when family members on the donor side and coaches, teachers and support staff on the receiving side coordinate around a shared whole-person philosophy, transitions are more likely to support wellbeing, competence development and balanced progress across domains not because demands are removed, but because support structures are flexible, expectations are scaffolded, and performance pressure is temporarily reduced (Linnér et al., 2022).

A central yet often implicit feature of transition environments is the experience of psychological safety, whereby young people feel protected from harm, and confident that concerns or difficulties will be noticed and responded to within the system. The importance of such safety and continuity is underscored by contrasting evidence from elite sport contexts where transitions are poorly supported (McGlinchey et al., 2022). Qualitative research on youth football academies shows that players released from professional pathways often receive little or no aftercare, leaving them feeling abandoned, isolated, and unsupported during a highly vulnerable transition period, with negative consequences for wellbeing and identity development (McGlinchey et al., 2022). From a safeguarding perspective, psychological safety can be actively supported through club or program culture and organizational practices (i.e., coaching practices) that foster trust (Vella et al., 2024).

## Gaming and esports transition environments

Building on the concept of transition environments (Henriksen et al., 2024), we propose that gaming and esports activities could function as transition environments when intentionally designed to bridge donor (i.e., home) and receiving (i.e., gaming/esports) settings. In the instance that a young person has become stuck in a “sinkhole” of gaming (i.e., where their psychological needs are being met predominantly through video game and are disengaging from work, education or training), the donor setting could be characterized by solitary gaming at home. This isolation makes them a difficult person to reach. In such a situation, parents and caregivers play an important role in the donor setting, as they are typically best positioned to observe early patterns of withdrawal (e.g., deteriorating mental health, difficulties at school, intensive gaming; Baker and Bishop, 2015; Hilding et al., 2025) and can engage with their child's gaming interests before contact with formal support systems occurs. Qualitative evidence from youth gaming cultures indicates that when parents engage with gaming in a curious and non-judgemental manner, this can strengthen trust, reduce conflict, and increase young people's willingness to share both positive and negative experiences related to their gaming activities (Askeland et al., 2020; Hilding et al., 2025). From a transition environments perspective, such parental engagement functions as a key donor-side resource that can legitimize gaming as a meaningful interest rather than a problem behavior. Thus, having support at home and from parents could play a key role in helping a young person transition from gaming in relative isolation into a physical community such as a gaming or esports club or program. However, from a transition environment perspective, this is also partially dependent upon the receiving setting.

In the receiving setting of the transition environment (i.e., structured gaming or esports clubs or programs embedded within existing educational, youth, or sport infrastructures; European grassroots esports, n.d.), adult facilitators (i.e., coaches, social workers, teachers and parents) can provide a sense of safety and valued belonging crucial for keeping young people engaged and motivated to remain engaged (Robinson and Aronica, 2015). If participation is sustained, young people may begin to experience psychological need fulfillment through gaming in a physical setting. Evidence suggests that receiving gaming and esports settings can support the development of academic, social, and interpersonal skills that extend beyond gameplay and may address early drivers of disengagement and marginalization (Nielsen and Hanghøj, 2019; Steinkuehler and King, 2009). Within effective receiving settings, young people can also be supported in developing internal and external resources, including self-regulation and social support, which contribute to improved coping (Freeman and Wohn, 2017; Sjödén and Trotter, 2024; Trotter et al., 2023). Importantly, longitudinal research from school-based esports programs indicates that participation in structured esports does not negatively impact self-regulation, physical activity, or perceived health when compared to non-participating peers (Trotter et al., 2022). Taken together, these findings suggest that as a receiving setting gaming- and esports-based clubs or programs offer a low-threshold means of engaging young people who are difficult to reach through traditional systems. Notably, participation in gaming and esports is strongly gendered, with boys and young men disproportionately represented, suggesting that such settings may be particularly well positioned to engage a group that is both overrepresented in esports and at elevated risk of disengagement from education and organized activity (Rogstad, 2021; Seidler, 2024). However, efforts should be made to ensure to identify how these environments can be designed to reduce identify-based barriers and inclusivity among girls and underrepresented groups.

From an ecological transition perspective, the effectiveness of gaming- and esports-based transition environments depends not on the donor or receiving setting in isolation, but on the quality of their interaction (Henriksen et al., 2024). In this view, transitions emerge through coordination, communication, and continuity between donor and receiving settings rather than through movement between discrete locations or programmes (Henriksen et al., 2024). In the context of youth gaming, parents and caregivers constitute a central donor setting, shaping young people's engagement through their attitudes, perceived control, and willingness to support or legitimize gaming as a meaningful activity. Evidence indicates that parental uncertainty and limited knowledge about esports can constrain support, even when parents are motivated to act in their child's best interests (Svensson et al., 2024). Receiving settings therefore play a critical role not only in supporting young people directly, but also in scaffolding the donor setting by providing parents with information, structure, and reassurance regarding norms, expectations, and developmental goals for both young people and their parents. Crucially, a unique potential of gaming and esports transition environments is that the first step in a transition can be digital, for example playing with other members of the club or program online, before meeting them in person. Digital engagement can provide a lower-threshold entry point, particularly for young people who experience social anxiety, are neuro-divergent, or have had prior negative experiences in organized sport or school contexts, allowing families to familiarize themselves with the other club or program members, routines and values before attending in-person sessions (Hilding et al., 2025). By reducing uncertainty and supporting predictability, such digitally mediated pathways can strengthen continuity across settings and support sustained engagement. However, it is important that when designing interventions based on this approach, adaptions to local cultural and contextual factors will be important. A clearer understanding of gaming and esports transition environments, is therefore central to designing programs capable of supporting young people (particularly those at greater risk of disengagement or who are neuro divergent) through key life transitions, while supporting their participation in society (Henriksen et al., 2024; Hilding et al., 2025; Svensson et al., 2024).

To date, gaming and esports have not been empirically examined as transition environments for young people disengaging from education or broader societal participation. This absence is not surprising. The transition environment concept itself is new, and existing applications have primarily focused on career transitions within high-performance sport rather than on those in the broader society (Henriksen et al., 2024). The present paper therefore advances a conceptual proposition rather than a validated model of practice. We suggest that the concept of transition environments holds promise for understanding how structured gaming and esports might function as bridging systems for disengaged youth, but systematic empirical work is needed to understand these environments in detail. Future research should examine how donor and receiving settings interact in practice, which transition preconditions (e.g., designated human and financial resources), established links between donor and receiving settings, structured transition support processes, whole-person philosophies, and overlaps in cultural practices are associated with sustained engagement and transition effectiveness. Only through such work can best-practice principles for gaming-based transition environments be identified and responsibly scaled.
